# Notes of a Case in Which Hydrophobia Was Prevented

**Published:** 1833

**Authors:** Landon C. Rives

**Affiliations:** Cincinnati


ART. III.—*Notes of a case in which Hydrophobia was prevented.* By LANDON C. RIVES, M. D., of Cincinnati.

Hydrophobia, one of the most ancient diseases of which we have any authentic record, remains to the present time one of the most incurable. Invited to the task by the terrific agonies of the sufferer, not less than by the fatal character of the malady, physicians have applied themselves diligently to the discovery of some method of cure; and *for this purpose*, they have employed at different periods, almost every therapeutic agent known to the profession. Nauseants long continued, mercurials freely and copiously administered, arsenic, bleeding ad deliquium animi, the cold bath, submersion almost to drowning, belladonna, nux vomica and the whole tribe of narcotics and antispasmodics, cantharides internally and externally, with many other of the most active medicines, have been successively used and lauded; and as if to furnish further demonstration of the fallacy of medical observation, the most extravagant eulogies have been pronounced upon the mildest, and most inert articles, as olive oil and the water plantain. Not only has the regular pharmacopaia been exhausted in the search after nostrums for this malady, [illegible] a tentative and empirical practice, (far beyond what has [illegible]ailed in any other disease,) has added a long catalogue of [illegible] remedies, all of which, after an ephemeral existence of a few days or months, have been consigned, by the unerring judgement of experience, to merited neglect, if not to oblivion. It may still, therefore, be assumed as an indisputable fact, that hitherto science and empiricism have *equally* failed to elicit any successful method of treatment, and that hydrophobia, in spite of all attempts to bring it under subjection to medicine, maintains with resistless power, its empire over the lives of its unhappy victims. But fortunately for the cause of humanity, the prophylaxis of rabies is far more certain than its therapia. Sugery has brought its resources to the aid of medicine, and by their combined action, the disease, if promptly attended to, may be prevented with tolerable certainty. As an illustration of this proposition, I submit the following case to the profession. It has no novelty I am aware, and if it have any interest, it derives it *solely* from furnishing a confirmation of the success of a prophylactic treatment, which is already received as the most probable method of averting a malady, which when established, bids defiance to every remedy, and bring upon the unhappy patient unmitigated suffering, and ultimately death, with scarcely an exception.

Case.—I was called on Friday the 16th of November last, to visit Mrs. S——th, who was bitten on that day by a dog said to be mad. Upon my arrival, nearly two hours after the occurrence, I found her surrounded by her neighbors and considerably agitated. I was not able to procure any history of the dog. The only facts which I could collect were, that a strange dog had entered the kitchen where Mrs. S——th was employed; that upon being ordered out, he made his way into an adjoining room, when being pursued by Mrs. S——th, (who was prompted by maternal apprehensions for the safety of her children, supposed to be in the room,) he turned upon her and made a furious assault, in which he inflicted a severe wound upon her left shoulder, and another of less magnitude on the left temple, about an inch or an inch and a half from the external canthus of the eye, and nearly in a horizontal line with it. Besides these wounds, shewing *evidently* the marks of the teeth, the cuticle near the left elbow was abraded for the space of a square inch, supposed by Mrs. S——th to have been done by the dogs feet.

Uncertain as I was from this account, of the condition of the dog, I *yet* did not hesitate to recommend a decisive treatment. Nothing less than excision and cauterization could satisfy my notions of professional fidelity and responsibility, in the case of bite by a dog indisputably rabid; and no computation of chances which I was enabled to make, founded upon the presumption that the dog was not in that state, seemed to relieve me from the obligation of proceeding as though the madness were ascertained. I accordingly recommended excision of the bitten part on the shoulder, a longitudinal and transverse incision down to the bone, through the wound in the temple, and cauterization with nitrate of silver. The proposition was received with horror by many of the *good* neighbors, who were present, and one or two of them, even ventured to remonstrate against a cruelty so wanton and unneccssary. Nevertheless the good sense of my patient triumphed over feminine timidity, and the suggestions of false tenderness; and, refusing to hazard her life upon the chance of the dogs not being mad, she submitted to the operation already indicated, and which need not be more particularly described. The slight wound near the elbow was treated by the application of lunar caustic alone. The eschars began to separate in a few days, a process which was accelerated by soft emollient poultices. The ulcers thus established, suppurated kindly, and were kept in a state of maturation for several weeks by digestive ointments. No internal medicines were administered, except such as seemed to be indicated by the condition of the sores; and apart from the treatment already recited, no other prophylactic, except a careful attention to sustain a cheerful disposition, by inspiring confidence and hope.

My patient remains well, never having suffered any anomalous or unpleasant symptoms whatever.

The suspicions of hydrophobia, which attached to the dog acquired additional strength, as the circumstances connected with his history were progressively developed, until they were finally confirmed by the occurrence of indisputable rabies in many animals bitten by him. It appears that the dog had been turned loose on the morning of the accident, by the Rev. Mr. Kemper, who resides on the Walnut Hills, a short distance from this city. He had purchased him a few weeks previously from a butcher, and for the purpose of keeping him until he became accustomed to his new home, he was secured with a chain. I am informed by Mr. K. that he observed nothing peculiar about the dog while in his possession, except that he was uncommonly *noisy and restless*, which was attributed by Mr. K. to impatience of confinement. *No suspicions of hydrophobia entered the mind of Mr. K., for he tells me that the dog both eat and *drank* as usual, was tractable and docile, and even on the morning of his liberation *lapped water*, and manifested his obedience by returning to his kennel when ordered. As soon as he was unchained, he took his course in the direction of his late home, but in passing through the farm, bit two hogs, the property of Mr. K. which died hydrophobous, in less than a month.

The dog was next seen in the market on Fifth street, in this city, where he bit a horse of Mr. Getts, a farmer, living three miles in the country. This horse also became hydrophobous and was shot by his owner. The next account of the dog, places him at Mr. Mannery's on the western side of Mill Creek. Here he entered the house abruptly, but committed no outrage. His conduct, however, exhibited a strange compound of irritability and of gentleness; laying his head on the lap of Mrs. F. an old lady of the family, he seemed to invite as well as to be pleased with attention, but immediately afterwards, taking his station under a bed in the room, his temper appeared to be suddenly revolutionized, and he manifested a disposition to bite all who approached him, particularly the children; whereupon Mr. M. taking him by the chain (which had been left suspended to his

* "Dubancix has seen mad dogs drink without difficulty, and plentifully." And "Dr. Gilman speaks of a dog which was not deemed rabid, *because it eat and drank well;* but as it seemed indisposed, it was killed, though not before it had bitten a man, who fell a victim to hydrophobia.

neck by Mr. Kemper) led him out and secured him to a stake in the yard. But he annoyed the family so much by howling, that Mr. M. very soon determined to dismiss him, and after taking the collar from his neck, with a view of hastening his departure, he set two dogs upon him. These were bitten by him, and killed the next day by Mr. M. whose apprehensions had been awakened by hearing of the treatment to which Mrs. S——th had been subjected. In pursuing his way down Mill Creek, the dog next bit another, belonging to Isaac Mills, and two cows of Mr. Stabler. Mills' dog died hydrophobous on Monday 26th, ten days after the reception of the wound. Stabler's cows died rabid also; one in three weeks, and the other in six, notwithstanding the *unfailing* skill of a German charletan, who very kindly insisted upon taking charge of *my* patient, had been exerted to preserve them. The only other animal that was bitten, as far as my information extends, was a horse belonging to a Mr. Stricker; and it is remarkable, that with the exception of my patient, it is the only instance in which hydrophobia has not occurred, among all the animals that were bitten, and whose lives were spared long enough for the developement of the malady. The wound, which was inflicted on the lip, was well washed and cleansed, and covered with a stimulant dressing.

In reviewing the preceeding case, several reflections suggest themselves; and first in regard to the dog, it would appear if the circumstances related may be relied upon, (and I believe they may,) that an aversion to water, does not constitute a uniform symptom of rabies in the canine species, however invariably it may accompany the malady in the human subject; nor is *furious madness*, as is ordinarily believed, always a concomitant of the disease in the dog, more than in man.

In regard to the patient whose case has been related, the most interesting and prominent reflection connects itself with her safety. But it may be asked, is she even now secure after the lapse of six months? I answer, *unhesitatingly*, that I believe she is. I am not ignorant that cases have been reported, in which it is said that the disease has occurred after the lapse of a much longer time, but in the falibility of medical opinion and the close resemblance of *symptomatic* rabies, to which the bitten are equally liable with other persons, I find ample reasons for distrusting extravagant statements of this sort. And moreover in the present case, I can perceive no reason, why a poison so concocted, as to produce its effects in every other instance, in a period varying from ten days to six weeks, should lay dormant in this for more than six months. As in other cases of inoculated diseases, the constitution of my patient may have been insusceptible to the action of this morbid poison. But this is a source of fallacy, which must in the very nature of things attach to every case of prophylactic treatment on record; and can in no way invalidate the present, which must be regarded as an example among many others of the success of preventive measures, unless it should be divested of that character, by the occurrence, hereafter, of hydrophobia in my patient, an event, which should it unhappily take place, shall be promptly announced to the readers of the Journal.

*Cincinnati, June,* 1833.

